# Evaluation of Dietary Supplement Use in Wheelchair Rugby Athletes

**DOI:** 10.3390/nu10121958

**Published:** 2018-12-11

**Authors:** Robyn F. Madden, Jane Shearer, David Legg, Jill A. Parnell

**Affiliations:** 1 Department of Kinesiology, University of Calgary, Calgary, AB T2N 1N4, Canada; rmadd656@mtroyal.ca (R.F.M.); jshearer@ucalgary.ca (J.S.); 2 Department of Health and Physical Education, Mount Royal University, Calgary, AB T3E 6K6, Canada; dlegg@mtroyal.ca

**Keywords:** wheelchair rugby, Paralympic athlete, quadriplegic athletes, dietary supplements

## Abstract

Wheelchair rugby is a rapidly growing Paralympic sport; however, research remains predominantly in the realms of physiology and biomechanics. Currently, there is little investigation into nutrition and dietary supplement use among wheelchair rugby athletes (WRA). The aim of this study was to assess the types of dietary supplements (DS) used, the prevalence of usage, and the reasons for use among WRA. The secondary aim was to report utilized and preferred sources of nutritional information among this population. A valid, reliable Dietary Supplement Questionnaire was used to report supplement use and reasons for use. Male (*n* = 33) and female (*n* = 9) WRA were recruited at a national tournament and through emailing coaches of various Canadian teams. Dietary supplement usage was prevalent as 90.9% of males and 77.8% of females reported usage within the past three months with the most regularly used supplements being vitamin D (26.2%), electrolytes (19.5%), and protein powder (19.5%). The most common reason for usage was performance. The top sources of nutrition information were dietitian/nutritionist and the internet. Further investigation into DS use is needed to help create nutritional guidelines that are accessible to WRA and athletes with disabilities in general.

## 1. Introduction

Originally named “Murderball”, wheelchair rugby is the most rapidly growing wheelchair sport worldwide [1]. Invented in Canada during the 1970s [2], the sport provides a Paralympic alternative to wheelchair basketball and track and field events, since the latter sports were dominated by more mobile paraplegic athletes versus those with quadriplegia, which wheelchair rugby is designed for [1]. The sport is comprised of intense physicality, aggression, and full body contact [1,3] while requiring efficient wheelchair manoeuvrability skills [4]. Wheelchair rugby differs from other wheelchair sports in that players must be tetraplegic also known as quadriplegic [5]. Furthermore, participants are classified based on impairment level, ranging from 0.5 (most impaired) to 3.5 (least impaired) [6] and the classification points of a team on the court cannot exceed eight points [2]. Wheelchair rugby is considered an intermittent sport and is played in eight minute quarters [6]. It is recognized by the International Paralympic Committee as a summer sport [7] and has held medal status in the Paralympic Games since 2000 [2]. Athletes who compete in wheelchair rugby typically have a spinal cord injury, whereas, athletes in Paralympic sport, in general, can include other disability sports and disability types.

Despite the growth in popularity of Paralympic sport [8], and wheelchair rugby in particular, research has predominantly occurred within the physiological and biomechanical domains [9]. As a result, few studies have focused on performance nutrition and behaviours of athletes with disabilities [10] not to mention those with a spinal cord injury (SCI). This is a noteworthy omission, as sport nutrition in this demographic is complex and nutritional plans should be individually-tailored [11] and consider both health and performance. Furthermore, most athletes with physical impairments have lower overall energy requirements in comparison to their able-bodied (AB) counterparts [12], which may lead to micronutrient deficiencies [13]. Depending upon the micronutrient, deficiencies could result in increased risk of illness and injury [14], compromised immune systems [15], and fatigue [16]; all of which can jeopardize sports performance.

In an attempt to offset dietary deficiencies, dietary supplements (DS) are often incorporated into an athlete’s nutritional plan. For the purpose of this study, a DS can be defined as “a food, food component, nutrient, or non-food compound that is purposefully ingested in addition to the habitually-consumed diet with the aim of achieving a specific health and/or performance benefit” [17]. Currently, DS use in wheelchair rugby athletes (WRA) has not been explored and few studies have reported the types and prevalence of use in other athletes with physical impairments [8,10,18,19] in comparison to the plethora of literature available in AB athletes [20,21,22,23,24,25,26,27,28]. To our knowledge, only one study has reported reasons of use in athletes with an impairment [10]. In addition, it is important to know the reasons athletes use dietary supplements (i.e., medical, performance, etc.) to ensure they are not over or under-supplementing. Furthermore, it is vital to know which sources of information athletes are utilizing for DS information, with prior research suggesting that dietitians are the main source for athletes with physical impairments [8,10]. Lastly, understanding *preferred* sources of information can help ensure accurate nutritional information is accessible to these athletes.

Presently, there are no nutritional guidelines tailored for populations with physical impairments [29]; and in particular those with quadriplegia. As a result, these athletes are forced to default to AB recommendations as a proxy. Although athletes with physical impairments can follow these recommendations, notable physiological differences including less muscle mass, limited sweating response, and impaired bowel function may affect the amounts needed [29]. Given the lack of sports nutrition investigation in WRA, the primary purpose of this study was to assess the types of dietary supplements used, the prevalence of usage, and the reasons for use among this population. The secondary objective was to report preferred sources of nutritional information for wheelchair rugby athletes.

## 2. Materials and Methods

### 2.1. Participants

Male and female athletes, 18 years and older, who met the criteria for an athlete with a physical disability, as set out by the International Paralympic Committee (IPC) [30], were recruited from various teams during the 2018 Canadian Wheelchair Rugby National Championships tournament or online via emailing various coaches of Canadian teams. Athletes who complete in wheelchair rugby are defined as those with impaired muscle power, impaired passive range of movement, limb deficiency, hypertonia, ataxia, or athetosis [7] and thus were eligible to participate in the study. Exclusion criteria included athletes who did not speak English or who were classified as having an intellectual impairment, as the questionnaires were not validated for these populations. The one exception to these exclusions was if English was an athlete’s second language. In this case, they were eligible to participate, if they confirmed that they understood the question or had a translator present. The study was approved by the University of Calgary Conjoint Faculties Research Ethics Board (Ethics ID: REB18-0236). Athlete written consent was provided prior to completing the Dietary Supplement Questionnaire.

### 2.2. Dietary Supplement Questionnaire

A modified version of a dietary supplement questionnaire [8,25,26] (Supplementary Files: S1 Dietary Supplement Questionnaire) was used to assess supplement intakes, reasons for use, and sources of supplement information. First, it identified the athlete’s age, gender, weight, height, sporting event, classification (0.5–3.5), current training phase, top level of competition, and hours of training per week. The questionnaire asked athletes if they took dietary supplements (i.e., yes or no) and to rank their overall diet as “not very healthy”, “pretty healthy (average)”, and “very healthy”. Athletes were also encouraged to describe the nature of their impairment (injury or medical condition). Second, the survey requested quantified supplement frequencies, reasons for using each individual supplement, and sources of information athletes used to receive information about supplements. Athletes were also asked about their preferred sources of nutritional information. Supplement usage was assessed using a list of 28 supplement classes including vitamins, minerals, fortified and sport beverages, electrolytes (sport or electrolyte drinks or supplements), protein powders, sport (carbohydrate-dense or protein-dense) bars and gels, buffers (i.e., sodium bicarbonate), fatty acids, plant extracts, and probiotics. If a supplement was not listed, space was provided to add it. Athletes indicated how often they used each type of supplement, using three options: regularly (at least 2 times per week), at times (you’ve tried it or only use it at certain times like during competition or when sick), and never (you have not tried it or you don’t know what this is) [8]. If an athlete indicated usage of a supplement, they were encouraged to provide the brand name and dosage beside it.

### 2.3. Procedures

Members of the research team attended the tournament and provided athletes with either a hard or electronic copy of the Dietary Supplement Questionnaire to fill out in a face-to-face manner. Two participants were recruited through emailing their coaches and filled out the online version of the questionnaire outside of the tournament. Members of the research team acted as scribes if a participant did not have the necessary dexterity to write or type.

### 2.4. Statistical Analysis

Data from the question ‘do you take any dietary supplements’ was categorized as yes or no. Dietary supplement use data were categorized into groups based on gender, age (≤30 years and 31 years and older), and sport classification (0.5–1.5 and 2.0–3.5). The age groups were based on dietary reference intake (DRI) values [31]. Sport classification groups were based on low pointers (0.5–1.5) and high pointers (2.0–3.5), which are characterized by differences in functional abilities and roles during a game [32]. Responses from the dietary supplement questionnaire were quantified as the percentage of respondents who consumed the supplement “Regularly”, “At Times”, or “Never”. Differences between gender, age, and classification groups were determined by a Pearson’s chi square test. The most common dietary supplements used overall were based on the total number of “Regularly” and “At Times” responses. The most common dietary supplements used by gender were based on the total number of “Regularly” scores. The reasons of use data were condensed into four categories: Performance, Medical/Health, Dietary, and Recommendation, and were quantified as the percentage of respondents who consumed the supplement. Performance reasons included ‘increase energy’, ‘increase endurance’, ‘improve exercise recovery’, ‘increase or maintain muscle mass, strength and/or power’, and ‘enhance overall athletic performance’. Medical/Health reasons include ‘medical’, ‘stay healthy’, ‘enhance immune system’, and ‘weight loss or weight gain’. Dietary reasons include ‘to improve your diet’, ‘enjoy the taste’, ‘convenient when hungry or thirsty’, ‘food allergy/sensitivity/intolerance’, and ‘special dietary needs’. Recommendation reasons include ‘someone told you to’, and ‘because others do’. If a participant put more than one reason, those reasons were condensed into the four categories. Sources of information by gender were analyzed using a chi square test. All analyses were performed using SPSS Statistics version 24 (IBM Corporation, Armonk, NY, USA).

## 3. Results

### 3.1. Participant Characteristics

A total of 42 athletes agreed to participate in the study and completed the questionnaires. Response rate to the individual questions ranged from 93% to 100%. Descriptive characteristics of the participants are outlined in Table 1.

Most of the athletes used at least one supplement over the course of the past three months (males 90.9%; females 77.8%, *p* = 0.281). When athletes were asked to rank their diet, 15.2% of males and 11.1% of females ranked their diet as “not very healthy”, 48.5% of males and 55.6% of females ranked their diet as “average”, and 36.4% of males and 33.3% of females thought their diet was “very healthy”. There were no differences between genders.

### 3.2. Dietary Supplement Use

**Figure 1:**
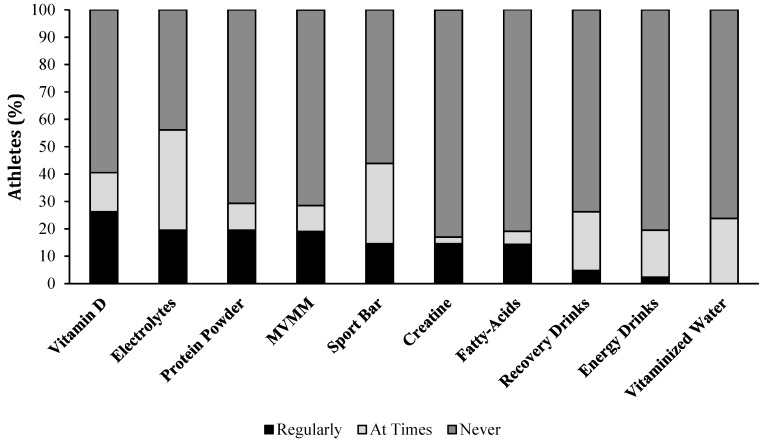
Dietary supplements commonly used by wheelchair rugby athletes. Data is presented in order of the most regularly used supplements. MVMM, multivitamin multi-mineral. Total numbers can be seen in Table 2.

Athlete use of dietary supplements is presented as those who consume the supplement regularly, at times, or never over the past three months (Figure 1).

Total supplement use and use by gender are provided in Table 2. The most common supplements based on ‘regular’ and ‘at times’ were electrolytes, sport bars, vitamin D, protein powder, and MVMM. Males most regularly used vitamin D, protein powder, and electrolytes, whereas females most regularly used MVMM and vitamin D. It is important to note that females did not ‘regularly’ use many dietary supplements; however, they were prominent in consuming supplements ‘at times’, including electrolytes, sport bars, and recovery drinks. Significant differences between genders were found in magnesium (*p* = 0.019) and probiotic pills (*p* = 0.022). Further, when the statistics were conducted by age group, athletes aged 31 years or older regularly used protein or sports bars 16.1% versus 10.0% in younger athletes. Conversely, younger athletes used bars occasionally 60.0% of the time versus 19.4% in the older group (*p* = 0.048). No other significant differences were seen in age group and no differences were seen in classification.

### 3.3. Reasons for Use

Reasons for use are presented as the number and percentage of participants who took a supplement for a specific reason (Table 3). Performance was the most frequent reason for supplementation (*n* = 67), followed by medical/health (*n* = 64), dietary (*n* = 28), and recommendation (*n* = 3).

### 3.4. Sources of Information

Sources of information are provided in Table 4. Males’ top utilized sources of dietary supplement information were dietician/nutritionist (51.5%), the internet (33.3%), and sport/fitness trainer (30.3%), whereas females’ sources were teammates/friends (44.4%) and the internet (33.3%). Two participants reported that they receive information from ‘research’ and ‘self’.

When the question was changed to “which way do you *prefer* to receive information about dietary supplements?” the most popular sources were individual nutrition consultation (dietitian), the internet (webpage/blogs), and coach/trainer (Table 5). Males’ top preferences were individual nutrition consultation (45.5%), the internet (27.3%), and coach/trainer (24.2%). Females’ top preferences were individual nutrition consultation (55.6%), the internet (33.3%), doctor/chiropractor/physiotherapist (22.2%), and coach/trainer (22.2%). No other preferred sources were listed; however, five athletes reported they were not interested in receiving information about dietary supplements.

## 4. Discussion

This research provides the first analysis of dietary supplement use in Canadian wheelchair rugby athletes. Furthermore, it is novel in that it directly links the individual supplement choice to a reason for use.

### 4.1. Dietary Supplement Usage

The WRA demonstrated a high usage of dietary supplements with 84.4% of the participants reporting using at least one DS over the past three months. This allows us to develop a benchmark as few studies have reported DS usage among athletes with disabilities [8,10,18,19]. One study investigating usage over five impairment types revealed 58% of athletes took at least one nutritional supplement in the previous six months [10]. Usage in the current study was higher; however, these results are in alignment with another study in Canadian athletes with a physical impairment [8]. Dietary supplement use in athletes of wheelchair sports may be beneficial given their reduced energy intakes [13], which, combined with a physical impairment, may contribute to micronutrient deficiencies [13]. Another factor potentially influencing usage is the training phase or season. The majority of the participants in the current study were in a competition phase (88.1%) and a high DS usage was reported. Divergent results were found by Krempien and Barr [19], as they reported that 44% of athletes with a SCI consumed supplements at home vs. 34% when at a training camp. In able bodied athletes, Erdman et al. [33] reported that DS usage was higher during the athletes’ training phase (93.1%) versus their competition phase (84.1%). Further investigation into supplement use during specific training phases may provide better insight into usage patterns as they relate to training periodization.

### 4.2. Dietary Supplement Types

The most regularly used dietary supplement was vitamin D. This finding coincides with the current literature, which reports frequent use of this vitamin in athletes with a physical impairment generally and those with a SCI specifically [8,34]. Maintaining sufficient levels of this micronutrient is important, as it has implications for athletic performance, including functionality of the skeletal muscles and maximal oxygen uptake [13]. Vitamin D deficiency can occur even in individuals without impairment [35] and supplementation of 1000 IU/day is often recommended in Canada [36]. Athletes with physical impairments possess a heightened deficiency risk compared to their AB counterparts due to potentially inadequate energy intakes [13]. Exposure to sunlight may not be feasible for some athletes with a SCI, as impaired thermoregulatory abilities can make skin hyper-sensitive when exposed to sunlight [14]. The coupling of vitamin D supplementation and monitoring levels in Para athletes, especially those competing in indoor sports and living in northern environments, is recommended [8,13].

Among the top supplements used were electrolyte drinks/supplements and protein powder. These findings are similar to studies that reported medication and supplement use at the 2004 Paralympic Games [18] and findings in elite, Canadian able-bodied athletes [33]. Some athletes with a SCI have compromised thermoregulation, thus have reduced sweat responses below the lesion level, but heightened sweat above the lesion [37]. This physiological factor may contribute to the regular use of electrolytes in an attempt to maintain hydration.

In sport, protein has roles in the synthesis of contractile and metabolic proteins and contributes to structural changes in tendon and bone tissue [11]. Current protein recommendations for AB athletes range from 1.2 to 2.0 g/kg and differ depending on the nature of the exercise performed (i.e., endurance vs. resistance), current phase within a periodization program, athletic goals, nutrient requirements, energy expenditure, and food choices [11]. Athletes with physical disabilities, such as a SCI, typically use less muscle mass than their AB counterparts [38]; therefore, specific protein needs for these athletes is unknown. Recent studies investigating dietary intakes in Paralympic athletes report adequate protein intake, based on AB recommendations, are being consumed from food alone [8,39].

### 4.3. Reasons for Use

Understanding the reasons for consuming DS is important to ensure that athletes are taking supplements for valid reasons. Findings of this study suggest that the most common reason WRA took DS was for performance. Athletes are keen to improve performance through supplementation; however, little evidence exists to suggest supplementation may actually improve performance outside of established deficiencies, at least in able bodied (AB) athletes. Exceptions include caffeine [40] and carbohydrate/electrolyte replacements [41]. The finding as to motivation for consumption is congruent with a study in elite AB athletes, which revealed the top reason for taking DS was to ‘increase energy’ [42].

The reasons associated with medical health were also common among participants. A noteworthy theme in medical/health reasons emerged, as some WRA reported taking cranberry pills, citing ‘bladder’ (infections) as their reason. According to the literature, urinary tract infections are common in those with a SCI [43]; however, the efficacy of cranberry to treat or prevent urinary tract infections requires further study [44,45].

### 4.4. Sources of Information

Being aware of dietary supplement information sources WRA utilize is vital to ensure they are receiving sound advice and that future nutritional guidelines are accessible. Our findings reported dietitian/nutritionist as the most utilized source, which is both congruent [8,10] and divergent [46,47] with other Paralympic studies. Both male and female athletes indicated the internet as their second most utilized and preferred source, which is concerning due to the high risk of non-scientific information and lack or absence of regulatory controls when purchasing such products online [10]. Teammates/friends, and sport/fitness trainers were also top choices for both genders; however, this also increases the risk of being given inaccurate information. Athletes and trainers should undergo basic sports nutrition training to enhance their knowledge and awareness [8].

The study is limited in that if a scribe or a translator for language was used, their answers may not be as accurate as if they had answered on their own. Furthermore, a small sample size was used, and multiple comparisons were made with a 5% level of significance, thus there is a risk of false positives. Caution is advised when interpreting the findings; however, given the paucity of research in this area, these preliminary findings are of high value.

## 5. Conclusions

Dietary supplement usage in WRA and their reasons for supplementation were evaluated. A variety of supplements were utilized for primarily performance and medical/health reasons. Future studies should evaluate dietary intakes and physiological levels of nutrients to determine optimal supplementation strategies in WRA.

## Figures and Tables

**Table 1:** Descriptive Characteristics.

Descriptive Characteristics	All	Males	Females
Participants	42	33 (78.6%)	9 (21.4%)
Age, years	36.3 (9.5)	36.8 (9.2)	34.3 (10.6)
Weight, kg	n/a	74.5 (14.7)	58.5 (8.1)
Height, m	n/a	1.79 (0.08)	1.68 (0.07)
BMI, kg/m^2^	22.8 (4.1)	23.3 (4.1)	20.5 (3.3)
Classification			
0.5–1.5	22 (52.4%)	17 (51.5%)	5 (55.6%)
2.0–3.5	20 (47.6%)	16 (48.5%)	4 (44.4%)
Level of Competition			
Provincial	5 (11.9%)	4 (12.1%)	1 (11.1%)
National	24 (57.1%)	18 (54.6%)	6 (66.7%)
International	13 (31.0%)	11 (33.3%)	2 (22.2%)

Descriptive characteristics are mean (standard deviation). BMI, body mass index, n/a, not applicable.

**Table 2:** Supplement use by Gender.

Supplement	Total *n* (%)	Male Regularly *n* (%)	Female Regularly *n* (%)	Male at Times *n* (%)	Female at Times *n* (%)	*p*
MVMM	12 (28.6)	6 (18.2)	2 (22.2)	4 (12.1)	0 (0.0)	0.544
Vitamin B	4 (9.5)	3 (9.1)	0 (0.0)	0 (0.0)	1 (11.1)	0.106
Vitamin C	6 (14.3)	2 (6.1)	1 (11.1)	3 (9.1)	0 (0.0)	0.582
Vitamin E	2 (4.8)	1 (3.0)	0 (0.0)	1 (3.0)	0 (0.0)	0.751
Vitamin D	17 (40.5)	9 (27.3)	2 (22.2)	5 (15.2)	1 (11.1)	0.883
Iron	2 (4.8)	1 (3.0)	1 (11.1)	0 (0.0)	0 (0.0)	0.313
Calcium	6 (14.3)	2 (6.1)	0 (0.0)	3 (9.1)	1 (11.1)	0.745
Magnesium	3 (7.1)	1 (3.0)	0 (0.0)	0 (0.0)	2 (22.2)	**0.019**
Vitaminized Water	10 (23.8)	0 (0.0)	0 (0.0)	10 (30.3)	0 (0.0)	0.058
Protein Powder	12 (29.3)	8 (25.0)	0 (0.0)	2 (6.3)	2 (22.2)	0.124
Protein or Sport Bars	18 (43.9)	6 (18.8)	0 (0.0)	9 (28.1)	3 (33.3)	0.371
BCAA	4 (9.5)	2 (6.1)	0 (0.0)	1 (3.0)	1 (11.1)	0.468
Glutamine	1 (2.4)	1 (3.0)	0 (0.0)	0 (0.0)	0 (0.0)	0.597
Buffers	1 (2.4)	0 (0.0)	0 (0.0)	1 (3.0)	0 (0.0)	0.597
Fatty-Acid Preparations	8 (19.0)	6 (18.2)	0 (0.0)	2 (6.1)	0 (0.0)	0.260
Sport or Electrolyte Drinks	23 (56.1)	8 (25.0)	0 (0.0)	11 (34.4)	4 (44.4)	0.246
Energy Drinks	8 (19.5)	1 (3.1)	0 (0.0)	7 (21.9)	0 (0.0)	0.247
Caffeine Pills	4 (9.5)	0 (0.0)	0 (0.0)	4 (12.1)	0 (0.0)	0.272
Pre-Workout Supplement	4 (9.8)	0 (0.0)	0 (0.0)	3 (9.4)	1 (11.1)	0.877
Creatine	7 (17.1)	6 (18.8)	0 (0.0)	1 (3.1)	0 (0.0)	0.305
Recovery Drinks	11 (26.2)	2 (6.1)	0 (0.0)	6 (18.2)	3 (33.3)	0.501
Plant Extracts/Herbal Supplements	6 (14.3)	2 (6.1)	0 (0.0)	3 (9.1)	1 (11.1)	0.745
Probiotic Pills	3 (7.3)	1 (3.1)	0 (0.0)	0 (0.0)	2 (22.2)	**0.022**
Sport Gels	2 (4.8)	0 (0.0)	0 (0.0)	2 (6.1)	0 (0.0)	0.449
Gummy/Bean	3 (7.1)	0 (0.0)	0 (0.0)	2 (6.1)	1 (11.1)	0.602

Intakes are presented as the number of athletes (%) who consume a dietary supplement regularly (i.e., at least twice per week) or at times (i.e., tried it or only use it at certain times such as during competition or when sick). Differences between genders were determined using a chi square test. *p* < 0.05 was considered significant. Athletes did not report using glucosamine, beta alanine, or beet root. Significant differences are bolded. MVMM, multivitamin multi-mineral. BCAA, branched chain amino acids. n/a, not applicable.

**Table 3:** Supplement Reason Frequencies.

Supplement	Performance *n* (%)	Medical/Health *n* (%)	Dietary *n* (%)	Recommendation *n* (%)
MVMM *	2 (15.4)	10 (76.9)	1 (7.7)	0 (0.0)
Vitamin B	0 (0.0)	4 (100.0)	0 (0.0)	0 (0.0)
Vitamin C	0 (0.0)	6 (100.0)	0 (0.0)	0 (0.0)
Vitamin E	0 (0.0)	2 (100.0)	0 (0.0)	0 (0.0)
Vitamin D	0 (0.0)	13 (92.9)	0 (0.0)	1 (7.1)
Iron	0 (0.0)	2 (100.0)	0 (0.0)	0 (0.0)
Calcium	0 (0.0)	4 (80.0)	0 (0.0)	1 (20.0)
Magnesium	0 (0.0)	2 (66.7)	0 (0.0)	1 (33.3)
Vitaminized Water	2 (28.6)	1 (14.3)	4 (57.1)	0 (0.0)
Protein Powder	4 (36.4)	5 (45.5)	2 (18.2)	0 (0.0)
Protein or Sport Bars	8 (47.1)	1 (5.9)	8 (47.1)	0 (0.0)
BCAA	4 (100.0)	0 (0.0)	0 (0.0)	0 (0.0)
Glutamine	0 (0.0)	1 (100.0)	0 (0.0)	0 (0.0)
Fatty-Acid Preparations	1 (12.5)	3 (37.5)	4 (50.0)	0 (0.0)
Sport or Electrolyte Drinks	13 (61.9)	2 (9.5)	6 (28.6)	0 (0.0)
Energy Drinks	4 (80.0)	0 (0.0)	1 (20.0)	0 (0.0)
Caffeine Pills	4 (100.0)	0 (0.0)	0 (0.0)	0 (0.0)
Pre-Workout Supplement	4 (100.0)	0 (0.0)	0 (0.0)	0 (0.0)
Creatine	7 (100.0)	0 (0.0)	0 (0.0)	0 (0.0)
Recovery Drinks	9 (81.8)	0 (0.0)	2 (18.2)	0 (0.0)
Plant Extracts/Herbal Supplements	0 (0.0)	6 (100.0)	0 (0.0)	0 (0.0)
Probiotic Pills	0 (0.0)	2 (100.0)	0 (0.0)	0 (0.0)
Sport Gels	2 (100.0)	0 (0.0)	0 (0.0)	0 (0.0)
Gummy/Bean	3 (100.0)	0 (0.0)	0 (0.0)	0 (0.0)

Dietary supplement reason frequencies. * One person put medical and performance reasons for taking MVMM and a “yes” response was placed in both categories. Buffers was not included, as athletes who responded “yes” did not provide a reason.

**Table 4:** Information Sources for Dietary Supplements.

Information Source	Number of Athletes *n* (%)
Internet (Websites, Facebook)	14 (33.3)
Sport/Fitness Trainer	11 (26.2)
Health Food Store	4 (9.5)
Product Labels	5 (11.9)
Pharmacist	7 (16.7)
Family	6 (14.3)
Print Media (magazines, books)	1 (2.4)
Workshops/Classes	0 (0.0)
Naturopath/Chiropractor	2 (4.8)
Television	3 (7.1)
Coach	3 (7.1)
Teammates/Friends	12 (28.6)
Medical Physician (Doctor)	9 (21.4)
Physio/Massage Therapist	2 (4.8)
Dietician/Nutritionist	19 (45.2)

Sources for information about dietary supplements.

**Table 5:** Preferred Information Sources for Dietary Supplements.

Preferred Information Source	Number of Athletes *n* (%)
Presentations	3 (7.1)
Family/Friends	7 (16.7)
Social media	4 (9.5)
Print media (pamphlets, books, magazines)	4 (9.5)
Coach/Trainer	10 (23.8)
E-mail	6 (14.3)
Individual nutrition consultation (Dietician)	20 (47.6)
Health food store/pharmacy	3 (7.1)
Internet (webpage, blogs)	12 (28.6)
Doctor/Chiropractor/Physiotherapist	6 (14.3)

Preferred sources for information about dietary supplements.

## Supplementary Materials

The following are available online at http://www.mdpi.com/2072-6643/10/12/1958/s1.
