# Supplementary material for: Evaluation of Dietary Supplement Use in Wheelchair Rugby Athletes
Source: Nutrients. 2018 Dec 11;10(12):1958. doi: 10.3390/nu10121958 (PMC6315401; doi:10.3390/nu10121958)
Supplement: Supplementary file 1 [file nutrients-10-01958-s001.pdf]

Dietary Supplement Questionnaire

Please ensure you sign the consent form prior to completing this questionnaire.

Athlete Name: ____

Age:____ **Date of Birth:**____________ **Gender: M / F** **Weight:** _________ **Height:**____________

Competitive Sport: ____________________________________ **Classification:** ___________________ (0.5-3.5)

Please describe the nature of your impairment (injury or medical condition):

____

____

____

What training phase/season is this for you?

☐ Competition ☐ Off-season training ☐ Off-season not training

Please indicate the top level of competition that you have competed in:

☐ Provincial (province you live in) ☐ National (within Canada) ☐ International (Countries other than Canada)

On average, how many hours do you train (exercise) each week?

☐ 0– 5 Hours/wk ☐ 6 – 10 Hours/wk ☐ 11-15 Hours/wk ☐ > 15 Hours/wk

How would you rate your diet (how healthy you eat everyday)?

☐ Not Very Healthy ☐ Pretty Healthy (average) ☐ Very Healthy

Dietary Supplement Use: Do you take any dietary supplements? (e.g. sport drinks like Gatorade, multivitamin, multimineral, vitamins/minerals, protein supplements, sport bars, energy drinks, herbal products, fish oil etc)

☐ Yes ☐ No

Dietary Supplement Choices

Consider your dietary supplement use over the last **3 months**. For each of the dietary supplements listed below, please indicate "How Often", "Supplement Name", and "Reason for Use".

How Often: Please place an "X" in the box that best describes how often you use the supplements listed. Your choices are "Regularly" (at least twice per week), "At Times" (you've tried it or only use it at certain times like during competition or when sick) or "Never" (you have not tried it or you don't know what this is).

Supplement Name: If you do take a supplement please write the exact one in the "Supplement Name" column if you know it.

Reason for Use (Please write the letter that corresponds to your reason or describe the reason if not listed)

- **A.** Medical (your doctor told you to)
- **B.** Increase energy (so you don't feel tired)
- **C.** Increase endurance (how long you can exercise)
- **D.** Someone told you to (coach, parent, friend)
- **E.** To improve your diet (food you eat everyday)
- **F.** Improve exercise recovery (after exercise)
- **G.** Because others (friends, family, teammates) do
- **H.** Enjoy the taste
- **I.** Stay healthy
- **J.** Enhance immune system (so you don't get sick)
- **K.** Convenient when hungry or thirsty
- **L.** Weight loss or weight gain
- **M.** Increase or maintain muscle mass, strength and/or power
- **N.** Enhance overall athletic performance
- **O.** Food allergy/ sensitivity/ intolerance
- **P.** Special dietary needs

**Dietary Supplement**	**How Often** (check box)			**Supplement Name**	**Reason for Use** (letter)
	**Regularly**	**At Times**	**Never**		
Multivitamin & mineral Pill					
Pill with just B Vitamins (e.g. Vitamin B12, Folic Acid, Vitamin B complex, Vitamin B6 Stress Tabs)					
Pill with just Vitamin C					
Pill with just Vitamin E					
Pill with just Vitamin D					
Other vitamin pills, please list in "Name" column					

Dietary Supplement	How Often			Name	Reason for Use
	**Regularly**	**At Times**	**Never**		
Pill with just Iron					
Pill with just Calcium					
Pill with just Magnesium					
Other minerals, please list in "Name" column					
Vitaminized Water (e.g. Vitamin Water, Aquafina Plus)					
Protein powder (e.g. whey/soy/hemp/rice)					
Protein or Sport Bars (e.g. Clif Bar, Power bar, Luna bar, Vector bar)					
Branched chain amino acids (BCCA)					
Glucosamine					
Beta-alanine					
Glutamine					
Beetroot Juice/Supplement					
Buffers (e.g. sodium bicarbonate, sodium citrate)					
Fatty-acid preparations (e.g. Flax seed oil, omega 3 oil or pill, fish oil or pill)					
Sport or electrolyte drinks/supplement (e.g. Gatorade, Powerade, Refresh, Nuun, G2, Salt tablets)					

Dietary Supplement	How Often			Name	Reason for Use
	Regularly	At Times	Never		
Energy drinks (e.g. Red Bull, Rockstar, Monster)					
Caffeine Pills does NOT include coffee (e.g. No Doz)					
Pre-Workout Supplement (e.g. Cellucor C4)					
Creatine (alone or in combination)					
Recovery Drinks (e.g. PureSport Recovery, CytoSport Protein Pure, Boost, Ensure, Breakfast Anytime)					
Plant extracts/Herbal Supplements (e.g. Echinacea, Cold FX, ginseng, garlic, oil of oregano, rose hip, turmeric)					
Probiotic pills NOT yogurt					
Sport Gels (e.g. Powergel, Clif shots, Carb- BOOM).					
Gummy/Bean (e.g. Sharkie, Clif Block, Sport Beans)					
Other (Any vitamin, mineral, herb, botanical, amino acid, dietary substance, concentrate, metabolite, constituent or extract not listed above.) Please list in the "Name" column					

Where do you get information about dietary supplements? Please check all that apply.

☐ Internet (Websites, Facebook)	☐ Pharmacist	☐ Naturopath/Chiropractor	☐ Medical Physician (Doctor)
☐ Sport/Fitness Trainer	☐ Family	☐ Television	☐ Physio/Massage Therapist
☐ Health Food Store	☐ Print Media (magazines, books)	☐ Coach	☐ Dietitian/ Nutritionist
☐ Product Labels	☐ Workshops/Classes	☐ Teammates/Friends	☐ Other (please list):

Which way do you prefer to receive information about dietary supplements? Please check all that apply.

☐ Presentations	☐ Print media (pamphlets, books, magazines)	☐ Individual nutrition consultation (dietitian)	☐ Internet (webpages/blogs)
☐ Family/Friends	☐ Coach/Trainer	☐ Health Food Store/Pharmacy	☐ Doctor/Chiropractor/Physiotherapist
☐ Social media	☐ E-mail	☐ Other: ______________	☐ Not interested in information

Thank you for taking the time to complete the questionnaire!
